# Obituary

**Published:** 1913-04-15

**Authors:** 


### OBITUARY.

Norman W. Kingsley, died at his home in Warren Point, N. J., February 20th, 1913. He was born October 26th, 1829, at Stockholm, N. Y. He began the study of dentistry with his uncle Dr. A. W. Kingsley at Elizabeth, N. Y., in 1848, when he was 19 years old. He began practicing for himself in Oswego, N. Y., in 1850, but moved to New York City in 1852 and became a partner with Dr. Solyman Brown. In 1865 he founded and became the first Dean and Professor of Dental Art and Mechanism. He invented the present style of making obturators for cleft palate and his book on Oral Deformities is the best work ever written on this subject. He also wrote the first work on Modern Orthodontia. Dr. Kingsley was an expert dental mechanic; a fine wood and ivory carver, a sculptor of note and did much commendable work as an artist especially in Pyrography. He traveled much and was well known abroad for his oral, surgical and dental skill. He enjoyed a large practice in New York for many years and was highly esteemed by his patients and the profession. He retired to a country home in the town of Warren Point, N. J., a few years ago where he died in his 84th year. He was a hard worker and a man of many talents which he cultivated as far as he could. He never seemed to have been a strong factor in professional politics, at least he was not a leader. He has left an important stamp on the profession which will keep his name prominent for many years.

